# Qushi Huayu decoction attenuated hepatic lipid accumulation via JAK2/STAT3/CPT-1A-related fatty acid β-oxidation in mice with non-alcoholic steatohepatitis

**DOI:** 10.1080/13880209.2022.2134898

**Published:** 2022-10-29

**Authors:** QinMei Sun, Xin Wang, Xin Xin, ZiMing An, YiYang Hu, Qin Feng

**Affiliations:** a Institute of Liver Diseases, Shuguang Hospital Affiliated to Shanghai University of Traditional Chinese Medicine, Shanghai, China; b Shanghai Key Laboratory of Traditional Chinese Clinical Medicine, Shanghai, China; c Key Laboratory of Liver and Kidney Diseases, Shanghai University of Traditional Chinese Medicine, Ministry of Education, Shanghai, China

**Keywords:** NASH, traditional Chinese medicine, lipid metabolism, hepatic steatohepatitis

## Abstract

**Context:**

Qushi Huayu decoction (QHD) has been clinically used for treating non-alcoholic steatohepatits (NASH). However, little is known about the effect of QHD on fatty acid β-oxidation (FAO)-dependent lipid consumption.

**Objective:**

To investigate the mechanism of QHD on FAO-related hepatic lipid accumulation.

**Materials and methods:**

Male C57BL/6J mice were randomly divided into 5 groups (*n* = 8): normal diet and drinking water (CON), high-fat and high-carbohydrate diet (HFHC), QHD-L (2.875 g/kg), QHD-H (11.5 g/kg) and obeticholic acid (OCA) (10 mg/kg/day) groups. All mice freely consumed an appropriate diet for 18 weeks, and QHD was orally administered in the last 6 weeks. Measurements of general condition, hepatic histopathology, and JAK2/STAT3 signalling pathway were taken.

**Results:**

QHD significantly improved NASH in mice, as reflected by improving serum glucolipid metabolism, decreasing enzymes activities, reducing hepatic triglyceride (HFHC: 70.07 ± 2.81 mg/g; QHD-H: 34.06 ± 5.74 mg/g) and ameliorating hepatic steatosis, inflammation in pathology. Further, both the mRNA and protein level of hepatic CPT-1A (*p* < 0.05), a rate-limiting enzyme of FAO, increased drastically following QHD treatment. Meanwhile, the content of hepatic ATP (*p* < 0.05) increased significantly after treatment with QHD. Further mechanistic results revealed that both the total protein and nuclear p-STAT3 in the liver were significantly down-regulated after QHD treatment. The protein level of hepatic p-JAK2 was significantly inhibited by QHD (*p* < 0.05 or *p* < 0.01).

**Conclusions:**

QHD could attenuate lipid accumulation by increasing JAK2/STAT3/CPT-1A-related FAO, which provides a scientific basis for the clinical application of QHD in treating NASH.

## Introduction

Non-alcoholic fatty liver disease (NAFLD), characterized by excessive lipid deposition and metabolic dysfunction in hepatocytes, has become the most common chronic liver disease worldwide (Brunt et al. 2015). The spectrum of histopathology ranges from simple steatosis (non-alcoholic fatty liver, NAFL) to non-alcoholic steatohepatitis (NASH). Available evidence indicates that the risk factors contributing to the evolution of NAFL to NASH involve lipotoxicity, insulin resistance, chronic liver inflammation, oxidative damage, mitochondrial dysfunction and endoplasmic reticulum stress (Tilg and Moschen 2010; Suzuki and Diehl 2017). Despite progress in this field, the pathogenesis of NASH remains unclear. Lipid accumulation plays a pivotal role in NASH (Monsenego et al. 2012). Lipid levels maintain a dynamic balance between fat synthesis and fat consumption, and unbalanced lipid metabolism leads to fat accumulation in the liver, resulting in hepatic steatosis. Therefore, accelerating fat metabolism is an effective way to reduce lipid accumulation in the liver (Koo 2013).

Mitochondrial fatty acid β-oxidation (FAO) is the primary mechanism responsible for fat consumption. Free fatty acids (FFAs) are esterified with coenzyme A, transported to the mitochondrial matrix, and oxidized to produce acetyl coenzyme A, which eventually undergoes β-oxidation (Dai et al. 2018). In FAO, carnitine palmitoyl transferase (CPT) systems mediate the transport of FFAs. These systems consist of three proteins: CPT-1, CPT-2 and acylcarnitine translocase. CPT-1, which is the rate-limiting enzyme of FAO, is responsible for the initial enzymatic reaction for FFA transport. Activation of CPT-1 promotes energy expenditure and has metabolic benefits (Shin et al. 2006). It is well known that mitochondrial dysfunction contributes to the onset of NAFLD because it affects liver lipid homeostasis and promotes lipid peroxidation, cytokine release and cell death (Begriche et al. 2006). Increasing evidence suggests that improving mitochondrial dysfunction is an effective strategy for reducing lipid overload. In conclusion, there is evidence that promoting FAO for the condition of hepatic fat accumulation has significant benefits.

The Janus Kinase 2 (JAK2)/signal transducer and activator of transcription 3 (STAT3) pathway not only regulates CPT-1 but also plays an important role in the regulation of lipid metabolism. Phosphorylated STAT3 (p-STAT3) acts on the promoter region of CPT-1 and inhibits its activity to reduce FAO. The relationship between the JAK2/STAT3/CPT-1 pathway and FAO has been well defined (Wang et al. 2018). Ectopic expression of STAT3 in the liver increases the circulating lipids in metabolic diseases by altering the expression of liver genes involved in hepatic lipid metabolism (Richard and Stephens 2011). JAK2/STAT3 activity was significantly increased in the livers of NASH mice, which contributed to the progression of steatosis (Zhu, Zhou et al. 2018). Therefore, the JAK2/STAT3/CPT-1A pathway may cause a reduction in hepatic lipid deposition.

Qushi Huayu Decoction (QHD), known to have robust therapeutic effects in treating NASH *in vivo* and *in vitro*, has been clinically used for decades (Feng et al. 2013; Leng et al. 2020). It contains five herbs, namely *Artemisia capillaris* Thunb (Compositae) (leaf), *Reynoutria japonica* Houtt (Polygonaceae) (rhizome and root), *Gardenia jasminoides* J. Ellis (Rubiaceae) (fruit), and *Hypericum japonicum* Thunb. ex Murray (Clusiaceae) (all), and *Rhizoma Wenyujin Concisum* (Zingiberaceae) (rhizome). Among the constituents identified in rodents, many prototype compounds were reported to have significant anti-NAFLD activities in previous study. For example, chlorogenic acid could prevent fatty liver by upregulating the expression of peroxisomal proliferator-activated receptor α (PPARα) (Wan et al. 2013). Quercetin was found to ameliorate NAFLD through the nuclear erythroid 2-related factor 2 (Nrf-2) pathway (Zhu, Xiong et al. 2018). Specifically, QHD reversed the FFA-induced decrease in the phosphorylation levels of adenosine 5′-monophosphate-activated protein kinase (AMPK) and acetyl-CoA carboxylase (ACC), and decreased the hepatic nuclear protein expression of sterol regulatory element binding protein-1 (SREBP-1) and carbohydrate response element binding protein (ChREBP) (Feng et al. 2013). As aforementioned, the role of lipid synthesis during the action of QHD on NASH has been discussed. However, whether FAO is involved in lipid consumption when QHD is used on NASH remains unclear.

In the present study, a HFHC-diet mouse model was established to determine the pharmacological effects of QHD on NASH and its associated molecular mechanisms *in vivo*. From the perspective of lipid consumption, the function of CPT-1A-related hepatic FAO in lipid metabolism was discussed. Then, we found that the JAK2/STAT3 signalling pathway probably contributes to the activation of CPT-1A. In general, QHD significantly increased JAK2/STAT3/CPT-1A-related FAO and prevented lipid accumulation in NASH.

## Materials and methods

### Animals

Male C57BL/6 mice (aged 7–8 weeks and weighed 20–22 g, *n* = 40) used in the present study were obtained from the Bikai Animal Experiment Co., Ltd. All mice were maintained in specific pathogen-free conditions with a 12 h light/dark cycle. All experimental procedures were performed in accordance with the National Institutes of Health Guidelines for Laboratory Animals and approved by the Animal Ethics Committee of Shanghai University of Chinese Medicine (Permission Number: ACU-29(20161230)).

### Preparation of QHD

QHD contains *Artemisia capillaris* (leaf), *Reynoutria japonica* (rhizome and root), *Gardenia jasminoides* (fruit), and *Hypericum japonicum* (all), and *Rhizoma Wenyujin Concisum* (rhizome). Qualified Chinese herbal medicine was purchased from Shanghai Yidaxing Pharmaceutical Co., Ltd. (Shanghai, China) and identified by Dr Zhixiong Li from Shanghai Institute of Materia Medica (Shanghai, China). The QHD was extracted according to the published literature. In brief, A. capillaries (120 g), *P. cuspidatum* (90 g), *C. longa* (60 g), *G. jasminoides* (60 g), and *H. japonicum* (90 g) were mixed and soaked together in 4200 mL of water for 1 h, and then boiled for 1.5 h. After the filtrate was collected, the residue was continued to be boiled twice in water. For each extraction, the volume of water was 3360 mL, and time was 1.5 h. Finally, all filtrates were mixed and concentrated to 150 mL, to get the QHD at 2.8 g crude drug/mL.

Based on the HPLC–Q-TOF of absorbed prototypes and generated metabolites in the portal vein plasma, liver (the target organ), and systemic plasma, a total of 66 constituents were identified in the QHD. Among these, 25 compounds were determined accurately by comparing with standards. There were six major structure types of ingredients in QHD (organic acids, iridoids, flavones, stilbenes, anthraquinones and naphthols) (Liu et al. 2020).

Among these, organic acid compounds comprised the largest amount, with a proportion of 38%, and included polyhydroxybenzoic acids, caffeoylquinic acids, and other polyphenols. Besides, more than 50% of the ingredients were glycosylated or sulphated conjugates, whose polarity were usually high.

### Animal experiments

After acclimation for one week, mice were randomly divided into five groups (*n* = 8 mice/group): (i) the control (CON) group, (ii) HFHC diet (HFHC) group, (iii) low-dose-QHD-treated (QHD-L) group, (iv) high-dose-QHD-treated (QHD-H) group, and (v) obeticholic acid (OCA)-treated group. Mice in the CON group were fed with the normal diet (10% kcal fat, D12450B; Research Diets, Inc, New Brunswick, NJ, USA) and normal drinking water, while mice in the other four groups were fed a high-fat (60% kcal fat, D12492i; Research Diets, USA) and high-carbohydrate (drinking water: 42 g/L, 55% fructose and 45% sucrose) diet. According to the conversion ratio between mice and humans (12.33), the daily QHD dose (g/kg) for mice can be calculated using the following formula: 12.33 × 56 g/60 kg = 11.5 g/kg (Huang et al. 2004). After 12 weeks of the respective diets, mice in the QHD-L group and QHD-H group received QHD at a dose of 2.875 and 11.5 g/kg, respectively, via gavage, whereas mice in the CON group and HFHC group received an equivalent amount of water via gavage. Mice in OCA group were also administered OCA (HY-12222; Medchem Express, Monmouth Junction, NJ, USA) intragastrically with a dose of 10 mg/kg/day. OCA has been proved to improve the pharmacodynamics on NASH in clinical and basic experiments. The treatment was administered for six weeks. At the end of the 18th week, the blood was collected, and the mice were sacrificed. Thereafter, the liver tissue was harvested and weighed for assay, and the liver index was calculated (liver index = liver weight/body weight).

### Haematoxylin-eosin staining and oil red O staining

To observe the histological changes, the formalin-fixed liver tissues were embedded in paraffin. Then, the sections (4 μm thick) were stained with haematoxylin and eosin (H&E; Nanjing Jiancheng Institute of Bio Engineering, Inc., Nanjing, China). Next, the NAFLD activity score (NAS) system was used to evaluate histological liver damage, which included steatosis, lobular inflammation and hepatocellular ballooning (Kleiner et al. 2005).

Liver tissue, which was embedded in optimal cutting temperature medium (Sakura Finetek, Torrance, CA, USA) and snap-frozen in liquid nitrogen, was sectioned (10 μm thick) and stained with Oil Red O (Sigma, St. Louis, MO, USA) to visualize the lipid droplets within the hepatocytes.

### Biochemical measurements

Abdominal aortic blood was centrifuged at 3,000 rpm for 20 min to obtain the serum for further analysis. Liver tissue homogenate was obtained by adding homogenate medium (900 µL) to 100 mg of liver tissue, followed by centrifugation of the mixture at 3,000 rpm for 10 min, and collection of the supernatant for further analysis. The serum levels of total cholesterol (TC), serum triglyceride (TG), serum low-density lipoprotein cholesterol (LDL-C), serum aspartate aminotransferase (AST) (C010-2), serum alanine aminotransferase (ALT) (C009-2) (Jiancheng, Nanjing, China) and liver TG (A0-10017; Dongou, Zhejiang, China) were evaluated according to the manufacturer’s instructions. Hepatic FFA (A042-2-1) and ATP (A095-1-1) (Jiancheng, Nanjing, China) levels were examined using an assay kit according to the manufacturer’s instructions. The content of serum insulin was examined via using a commercial kit (90080; Crystal Chem, Elk Grove Village, IL, USA). HOMA-IR = (fasting blood glucose × fasting insulin content)/22.5 (Haffner et al. 1997).

### Quantitative real-time polymerase chain reaction (q-PCR)

Total RNA was extracted using TRIzol reagent (TAKARA, Toyoko, Japan). The concentration and purity of RNA were detected using a Nanodrop ND-1000 spectrophotometer (Thermo Fisher Scientific, Waltham, MA, USA) at a wavelength of 260/280 nm. Total RNA (0.5 μg per reaction) was reverse-transcribed into complementary DNA using SuperScript II Reverse Transcriptase (Invitrogen, Waltham, MA, USA) according to the manufacturer’s instructions. PCR amplification was performed in duplicates on complementary DNA diluted five times using SYBR green dye (TAKARA, Toyoko) on a Step One Sequence Detection System (Applied Biosystems, Waltham, MA, USA). The following primers were used: sense primer A (5′-TTG GGC AAT CTG GGC ACT TT-3′) and antisense primer A (5′-CTT TGC CTC CCT TCT GCT CT-3′) for CPT-1; sense primer A (5′-AGA GGG AAA TCG TGC GTG AC-3′) and antisense primer A (5′-CAA TAG TGA TGA CCT GGC CGT-3′) for β-actin was used as the reference gene. The relative abundance of genes was calculated by using the 2^−ΔΔC^_T_ formula.

### Western blot

Total protein was extracted using radioimmunoprecipitation assay lysis buffer while nucleus protein was extracted using a Nuclear protein Extraction Kit (BB-3166-1, Bestbio, China). Liver tissue proteins were separated by 4%–20% gradient sodium dodecyl sulfate-polyacrylamide gel electrophoresis (SDS-PAGE) and transferred onto polyvinylidene difluoride (PVDF) membranes (Millipore, Billerica, MA, USA). Then, the membranes were blocked in a solution containing TBST (10 mM Tris-HCl, pH7.4, 150 mM NaCl and 0.05% Tween 20) and 5% bovine serum albumin at 25 °C for 1 h. Subsequently, the membranes were separately incubated overnight at 4 °C with GAPDH (10494-1-AP; 1:1000 dilution; Proteintech, USA), Lamin A/C (10298-1-AP; 1:1000 dilution; Proteintech, USA), JAK2(3230; 1:1000 dilution; Cell Signaling Technology, Danvers, MA, USA), phosphorylated JAK2 (p-JAK2; 3776; 1:1000 dilution; Cell Signaling Technology, USA), STAT3 (4904; 1:1000 dilution; Cell Signaling Technology, USA), p-STAT3 (9145; 1:1000 dilution; Cell Signaling Technology, USA) and CPT-1A (ab128568; 1:1000 dilution; Abcam, Cambridge, UK) antibodies. After washing with TBST buffer, the membrane was incubated at 25 °C for 1 h with horseradish peroxidase-conjugated anti-rabbit antibody (Ab9722; 1:5000 dilution; Beyotime Biotechnology, Shanghai, China). After washing with TBST buffer, signals were detected by using a chemiluminescence reagent (Millipore, Burlington, MA, USA), and imaged and quantified using a Qinxiang gel imaging system (Qinxiang Scientific Instruments Co., Ltd., Shanghai, China). Data are presented as the ratio of phosphorylated proteins to total proteins, and are expressed as the fold-change compared to the control group.

### Statistical analysis

All statistical analyses were presented using GraphPad Prism 7.0 (GraphPad Software, San Diego, CA, USA). Data were presented as mean ± standard deviation. Significant differences between the groups were determined using one-way ANOVA and Tukey was used to do the *post hoc* test. A value of *p* < 0.05 was considered to be statistically significant.

## Results

### QHD improved general conditions, glucose metabolism and lipid metabolism in NASH mice

**Figure 1.**
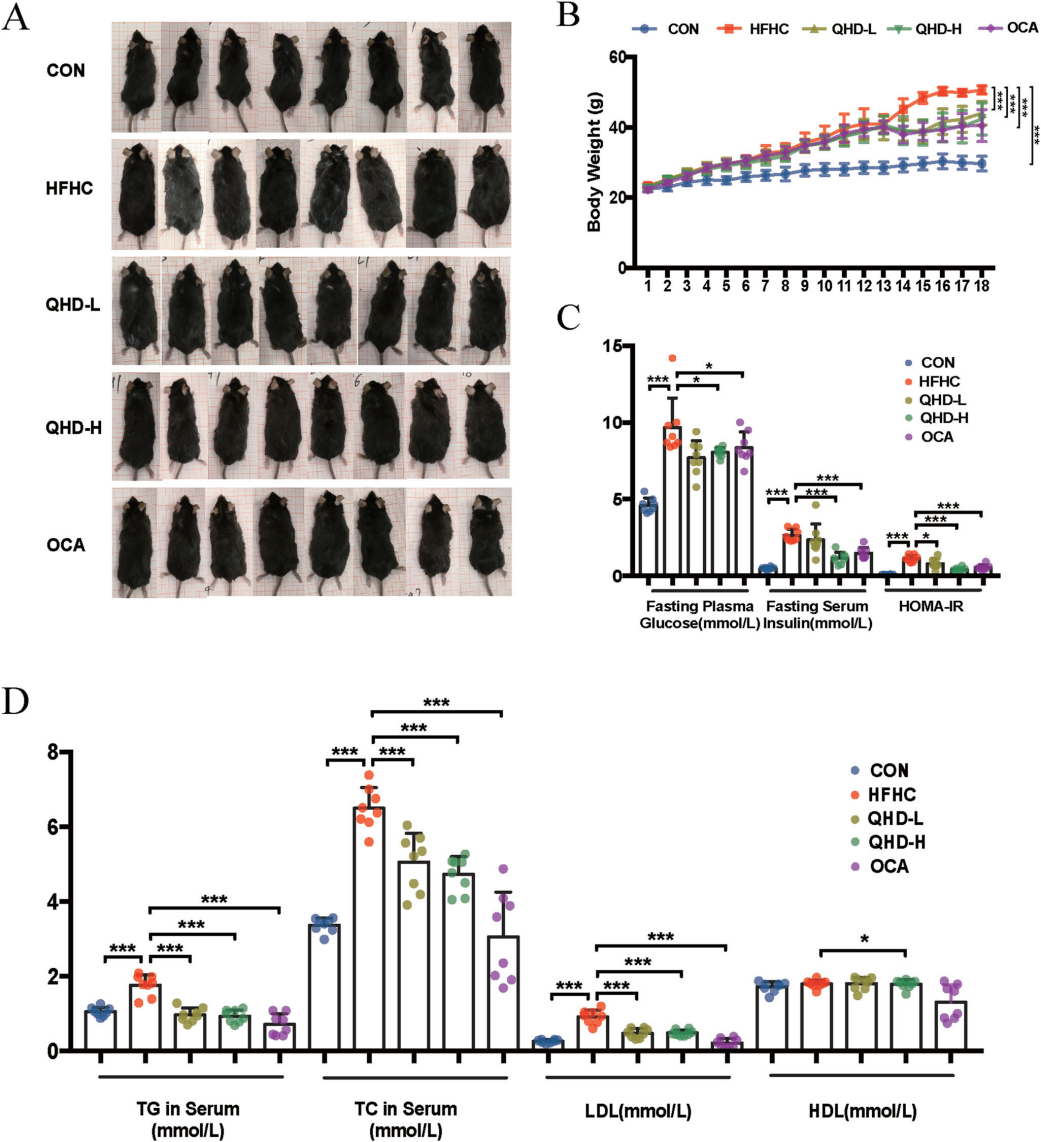
QHD improve general condition, glucose metabolism and lipid metabolism in NASH mice. (A) Mice morphology. (B) Body weight. (C) Fasting plasma glucose, insulin level and HOMA-IR in serum. (D) TG, TC, LDL and HDL level in serum. *n* = 8, All data are expressed as mean ± SD. *p < 0.05, **p < 0.01, ***p < 0.001.

To determine whether QHD could exhibit therapeutic potential, the overall condition of the mice, including body morphology and body weight at the end of the experiment, was observed. The mice in the HFHC group were significantly larger than those in the normal group. After treatment with QHD or OCA, the mice in the QHD-L, QHD-H and OCA groups were smaller than those in the HFHC group (Figure 1(A)). The body weight of the HFHC group was significantly higher than that of the CON group, revealing that an HFHC diet could lead to an increase in body weight. After treatment with QHD or OCA, the body weight of mice decreased significantly compared to that of the HFHC group (Figure 1(B)). There were no significant differences between the body weights of QHD-L and QHD-H groups. Overall, the OCA group mice had significantly reduced body weight compared with the HFHC group, although it did not differ significantly from that of the QHD-L and QHD-H groups.

After overnight fasting, blood serum was collected from the mice for measurement of serum biochemical parameters. Significantly increased fasting plasma glucose, fasting serum insulin and insulin resistance (in terms of the homeostatic model assessment for insulin resistance, HOMA-IR) were demonstrated in HFHC-fed mice compared with that of the CON group (*p* < 0.01) (Figure 1(C)). Meanwhile, enhanced content of TG, TC and LDL were also observed in HFHC-fed mice compared with those fed a normal diet (*p* < 0.01) (Figure 1(D)). After six weeks of QHD treatment, we observed a significant reduction in fasting blood glucose, fasting blood insulin, and HOMA-IR levels in mice in the QHD-H group (*p* < 0.01). In addition, the effect of QHD-L was less pronounced, but not significantly different from QHD-H (Figure 1(C)). In parallel, the contents of TG, TC and LDL were lower in the QHD-L and QHD-H groups than that in the HFHC group (*p* < 0.01) (Figure 1(D)). There were no significant differences between the two QHD groups (Figure 1(D)). Notably, the serum HDL levels in these groups did not show any discernable discrepancies.

### QHD ameliorated liver injury in NASH mice

**Figure 2.**
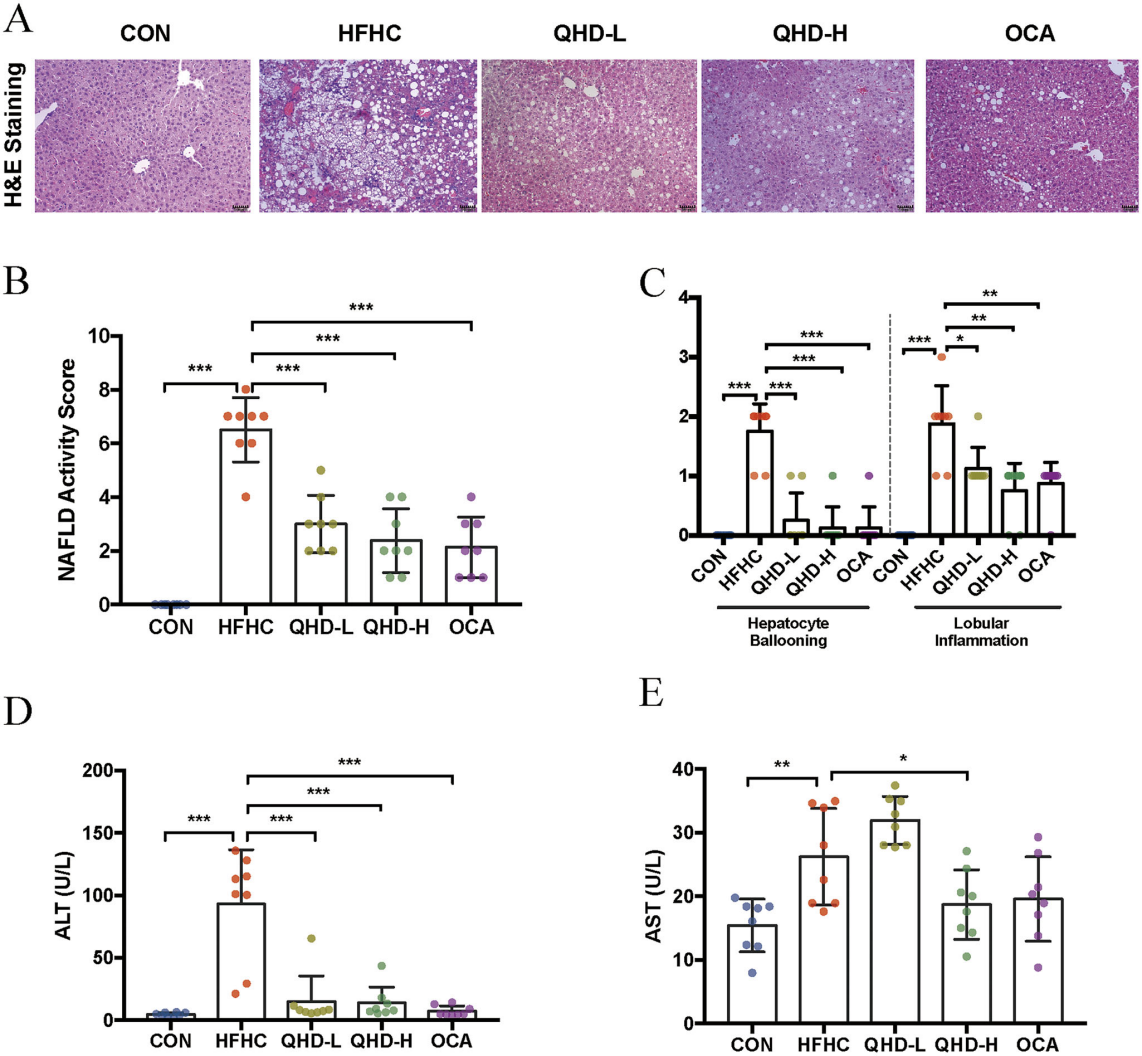
QHD ameliorates liver injury in NASH mice. (A–C) Representative H&E staining of liver tissue from mice showed histologic differences (200*). (D-E) ALT and AST level in serum. *n* = 8, All data are expressed as mean ± SD. *p < 0.05, **p < 0.01, ***p < 0.001.

Next, to further identify the effect of QHD on NASH, the histological changes and levels of hepatic ALT and AST activity were evaluated. H&E staining showed that large numbers of vacuoles and vesicles were found in the hepatocytes of mice with NASH (Figure 2(A)). The hepatocytes were pale and swollen, with sparse cytoplasmic edoema. Cytoplasmic Mallory-Denk bodies were found in the hepatocytes around the central vein. The degree of hepatic steatosis, lobular inflammation, and hepatic collagen regions, assessed using the NAS (Figure 2(B)), confirmed the increased severity of liver pathohistology in HFHC-fed mice (*p* < 0.01) (Figure 2(A–C)). In this study, different doses of QHD significantly improved hepatic histological changes in HFHC-fed mice. Meanwhile, consistent with the signs of histological inflammation, serum ALT and AST levels were significantly increased in HFHC-fed mice compared to those of the CON group (*p* < 0.01) (Figure 2(D)). After QHD treatment of HFHC-fed mice, a significant reduction in ALT activity was observed. Moreover, we also found that AST activity was significantly decreased in QHD-H-fed mice, but not in the QHD-L group compared to that in the HFHC group (*p* < 0.01) (Figure 2(E)).

### QHD attenuated lipid accumulation in NASH mice

**Figure 3.**
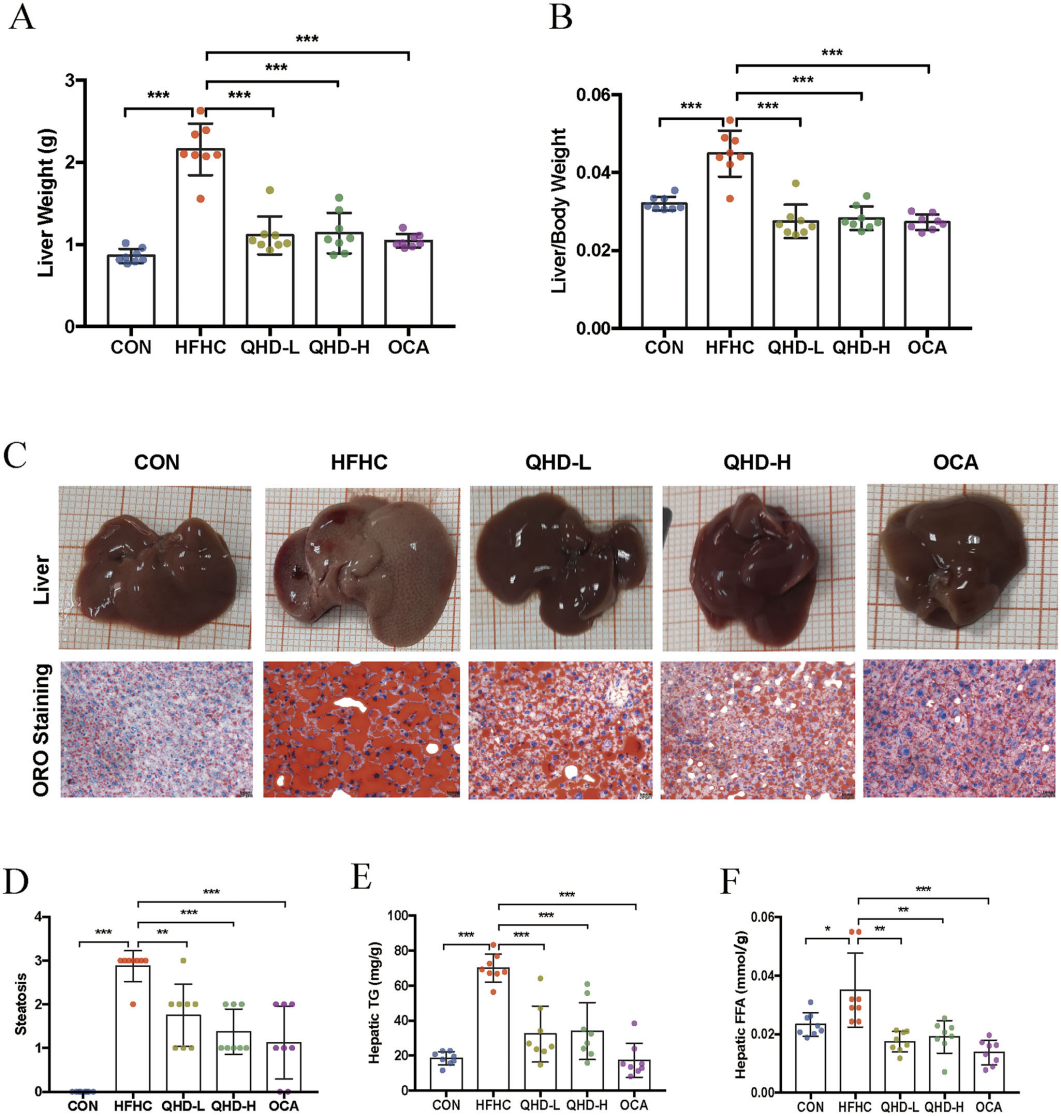
QHD attenuated lipid accumulation in NASH mice. (A) Liver weight. (B) Liver index. (C) Representative ORO staining of liver tissue from mice showed histologic differences (200*). (D) The score of steatosis in liver. (E) Hepatic TG level. (F) Hepatic FFA level. *n* = 8, All data are expressed as mean ± SD. *p < 0.05, **p < 0.01, ***p < 0.001.

To determine the effect of QHD on hepatic lipid accumulation in NASH mice, we first evaluated the changes in liver morphology in terms of liver weight and liver index. Our data showed that in the HFHC group, there was significantly enhanced liver weight and liver index compared with that of the CON group. QHD-treated mice had significantly lower liver weights and liver indices than mice in the HFHC group (*p* < 0.01) (Figure 3(A,B)). Lipid droplets were prominently attenuated after six weeks of QHD treatment (*p* < 0.01) as verified by Oil Red O staining and score of steatosis (Figure 3(C,D)). Furthermore, the hepatic TG and FFA contents of mice in the HFHC group were significantly higher than those in the CON group. Compared with the HFHC group, significantly decreased hepatic TG and FFA contents in mice of QHD-L and QHD-H groups were indicated (*p* < 0.01) (Figure 3(E,F)). Similarly, the pathological and biochemical amelioration was also observed in mice treated with OCA. Hence, QHD treatment markedly reduced hepatic lipid accumulation in HFHC-fed mice.

### QHD enhanced FAO in NASH mice

**Figure 4.**
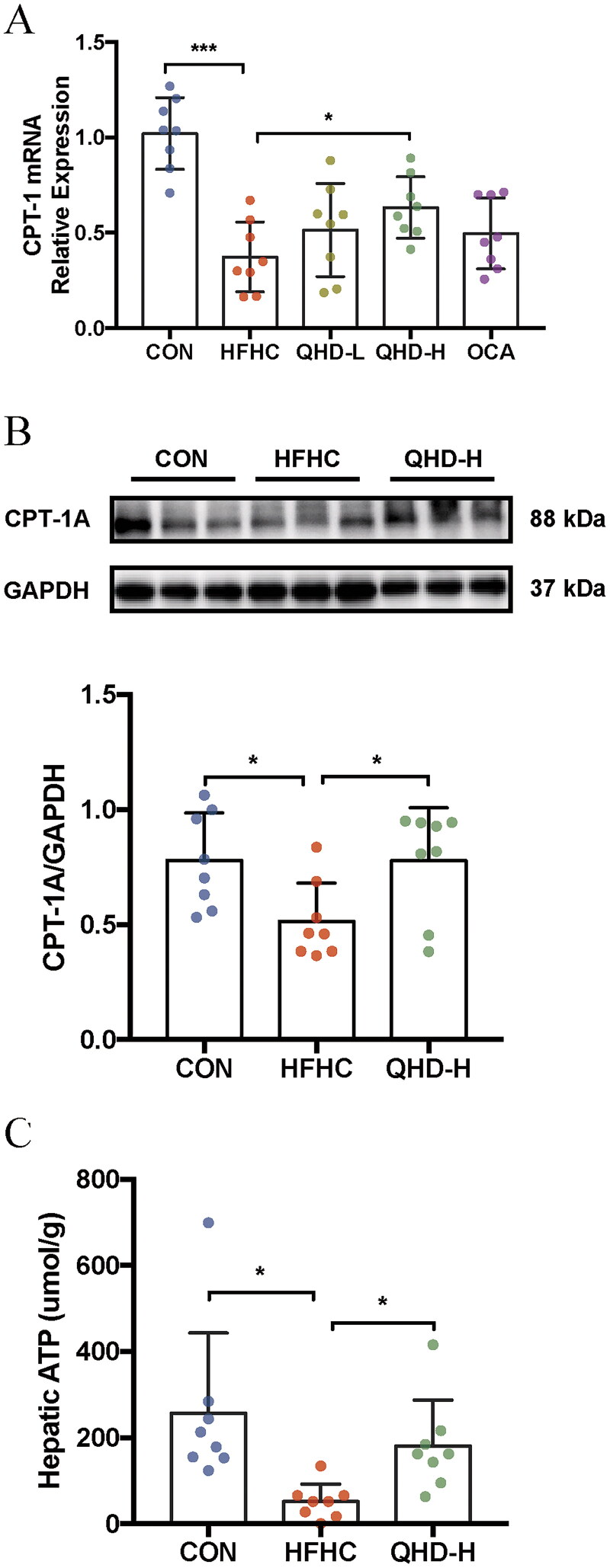
QHD increases Fatty Acid β-Oxidation in NASH mice. (A) Relative expression of CPT-1 mRNA level in liver tissue. (B) The protein levels of CPT-1A were detected by western blotting analysis. The samples derive from the same experiment and that gels/blots were processed in parallel (*n* = 8). (C) ATP level in liver. *n* = 8, All data are expressed as mean ± SD. *p < 0.05, **p < 0.01, ***p < 0.001.

To elucidate the potential role of FAO in lipid consumption in the presence of QHD, we measured the transcription and protein levels of hepatic CPT-1A in mice. CPT-IA, the crucial rate-limiting enzyme in FAO, was significantly downregulated at the transcription level in the liver tissue of HFHC-fed mice compared with that of mice in the CON group (*p* < 0.01 or *p* < 0.05) (Figure 4(A)). Again, a significant reduction in the level of CPT-1A was demonstrated in HFHC mice compared to that of CON group. After QHD treatment, we found that the mRNA and protein expression levels of CPT-1A were significantly enhanced in the QHD-H group mice compared to those in the HFHC group. Notably, increased mRNA levels of CPT-1A were only observed in QHD-H, but not in QHD-L mice (*p* < 0.01 or *p* < 0.05) (Figure 4(A,B)).

Therefore, the potential mechanism underlying the effect of high-dose QHD on FAO merits further discussion. ATP is produced in the mitochondria, which is not only the core of cell energy metabolism, but also the site of FAO generation. Hence, hepatic ATP content could indicate the level of cellular energy metabolism. In our data, QHD-H treatment significantly upregulated the expression of ATP in the liver of mice compared to that of the HFHC group (*p* < 0.01) (Figure 4(C)). These results suggest that the QHD treatment promoted FAO in HFHC-fed mice.

### QHD inhibited the phosphorylation levels of JAK2 and STAT3 in NASH mice

**Figure 5.**
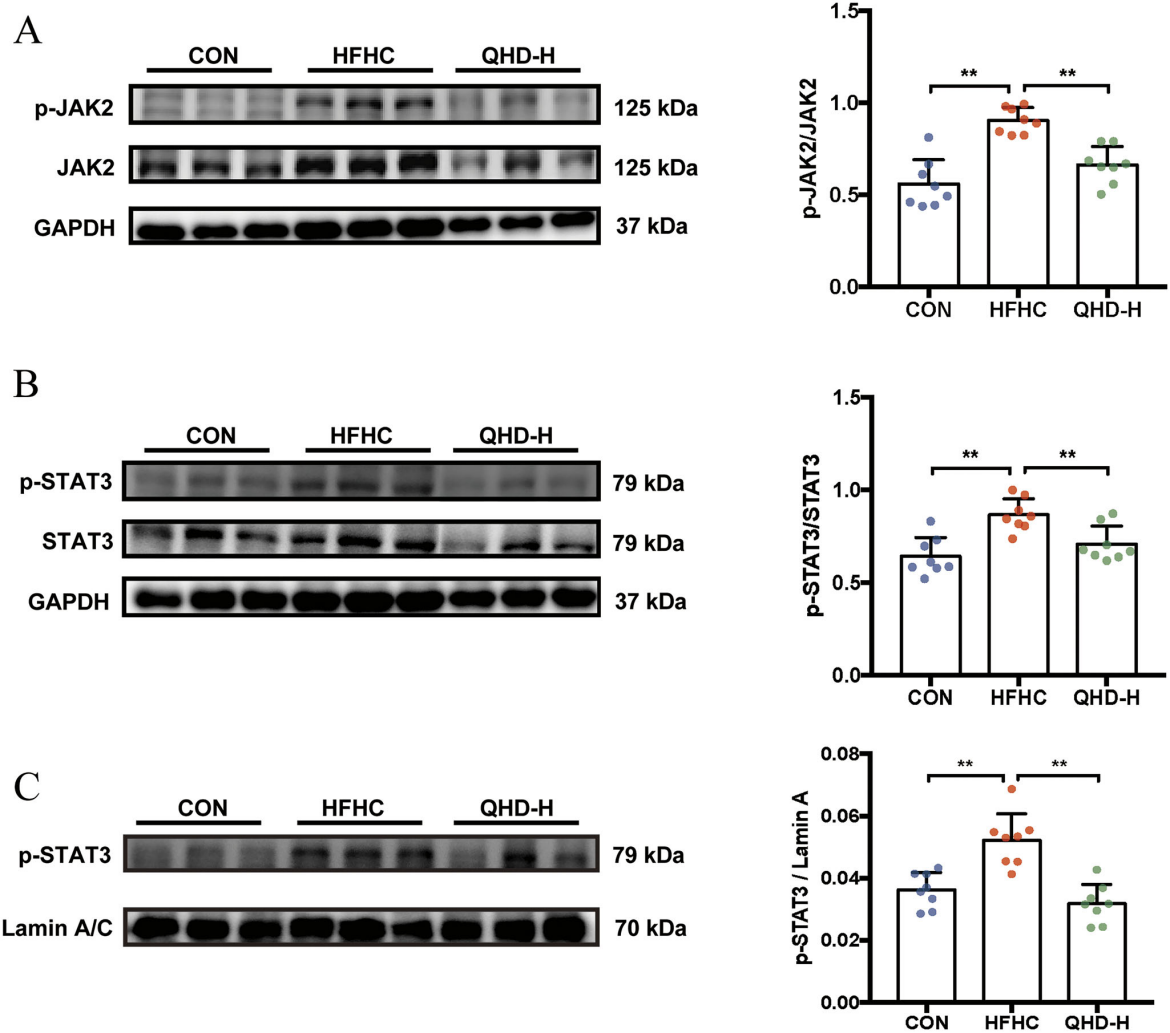
QHD inhibits the phosphorylation levels of JAK2 and STAT3 in NASH mice. (A,B) The phosphorylation levels of JAK2 and STAT3 in total protein were detected by western blotting analysis. (C) The level p-STAT3 in nuclear protein was detected by western blotting analysis. The samples derive from the same experiment and that gels/blots were processed in parallel. *n* = 8, All data are expressed as mean ± SD. *p < 0.05, **p < 0.01, ***p < 0.001.

The JAK2/STAT3 signalling pathway negatively regulates the transcription of CPT-1A during FAO under pathological conditions. To examine the role of JAK2/STAT3 on CPT-1A in QHD-treated mice, we measured the expression level of JAK2, p-JAK2, STAT3 and p-STAT3 in hepatic total protein, as well as the expression level of p-STAT3 in hepatic nuclear protein. In hepatic total protein, the results demonstrated that the HFHC diet remarkably enhanced the phosphorylation protein expressions of JAK2 and STAT3. Meanwhile, compared with mice in the HFHC group, a significant decrease in the phosphorylation of JAK2 and STAT3 proteins was observed in the QHD-H group (*p* < 0.01) (Figure 5(A,B)). Furthermore, in hepatic nuclear protein, QHD-H treatment significantly down-regulated the expression level of p-STAT3 compared to that of the HFHC group (*p* < 0.05) (Figure 5(C)).

In NASH mice, high-dose QHD showed a convincing upregulation of hepatic CPT-1A expression, thus triggering the FAO aggrandizement to ameliorate hepatic lipid accumulation. Furthermore, these may be related to the JAK2/STAT3 signalling pathway.

## Discussion

In the present study, we demonstrated that QHD is effective for the treatment of NAFLD *in vivo*. We first focussed on the effects of QHD on hepatic lipid accumulation in HFHC diet-induced NASH mice. Moreover, the pharmacological effects of two different doses of QHD on NASH were subsequently compared. After elucidating the improvement in liver pathology based on morphological observations and histological stains, we investigated the role of increased FAO on the therapeutic effect of QHD, namely accelerating hepatic lipid depletion, and highlighted that CPT-1A activation might inhibit the JAK2/STAT3 signalling pathway. Thus, we confirmed that QHD reduces hepatic lipid accumulation in the HFHC diet-induced NASH mice model by increasing FAO, which may be related to the JAK2/STAT3/CPT-1A pathway.

Hepatic lipid accumulation, a critical link in the pathogenesis of NASH, is considered to be caused by a variety of factors, including increased uptake of FFAs, impaired FAO and increased incidence of new fat generation (Donnelly et al. 2005; Postic and Girard 2008; Monsenego et al. 2012). Previous studies have shown that hepatic lipid accumulation is related to insulin resistance, mitochondrial stress, autophagy damage and endoplasmic reticulum stress, eventually leading to the progression of liver damage (Koo 2013). Therefore, reducing hepatic lipid accumulation is a vital and effective method for the treatment of NASH. In our previous study, four weeks of QHD treatment significantly reduced hepatic TG in NASH mice, thereby reducing lipid accumulation (Leng et al. 2020). Similarly, in rats fed with HFD diet, the lipid accumulation had significantly reduced after four weeks of QHD treatment. To be specific, it reversed the hepatic phosphorylation levels of AMPK and ACC, and subsequently decreased the nuclear protein expression of SREBP-1 and ChREBP (Feng et al. 2013). In this study, we used a well-documented NASH mouse model where NASH was induced by a high-fat and high-carbohydrate diet (Xin et al. 2020). Again, a continuous 18-week HFHC diet feeding significantly increased hepatic TG and FFA levels and induced severe insulin resistance. Predictably, six weeks of QHD treatment significantly reduced the levels of TGs and FFAs in the liver, and thus attenuated lipid accumulation in NASH mice.

Depending on the physiological conditions, increased FFAs have multiple metabolic destinations in the liver. Besides undergoing FAO, FFAs are esterified to TGs, packaged into very low-density lipoprotein (VLDL) via the condensation of cholesterol esters, phospholipids, and apolipoprotein (ApoB), and then released into the circulation. Otherwise, FFAs esterified into TG are stored in the form of fat droplets in hepatocytes (Browning and Horton 2004; Wolins et al. 2006). Evidently, of these metabolic fates of FFAs, FAO has received more attention as a mechanism underlying the reduction of FFAs in the liver. FAO plays a key role in metabolism and energy supply and is an extremely complex process. Generally, after fatty acids are transported across the plasma membrane, they are rapidly converted into acyl-CoA by acyl-CoA synthetases (Li et al. 2009; Houten and Wanders 2010; Rui 2014). Thereafter, acyl-CoA is converted to acylcarnitine by CPT-1 in the mitochondrial outer membrane. CPT-1 is the rate-limiting enzyme in FAO, which comprises three subtypes: CPT-1A (liver subtype), CPT-1B (muscle subtype) and CPT1C (brain subtype) (Bonnefont et al. 2004; Wolfgang et al. 2006). CPT-1A allows FFAs to enter the mitochondria, where it undergoes degradation into acetyl-CoA through β-oxidation. Impaired mitochondrial function results in severe disturbance of lipid metabolism, thus leading to serve lipid peroxidation and oxidative stress injury (Bartlett and Eaton 2004; Yin et al. 2011). Herein, 18 weeks of HFHC diet reduced the expression level of CPT-1A, thus decreasing the production of ATP in the liver, leading to severe mitochondrial dysfunction. Notably, these changes were significantly reversed by treatment with high-dose QHD, but not with low-dose QHD. The CPT-1 mRNA level in mice of QHD-L group had an increasing trend compared with that of HFHC group, though it was not statistically significantly different. Nevertheless, QHD-L still has a significant effect on NAFLD. As mentioned in the introduction, QHD has been proved to decrease hepatic lipid synthesis in our previous study. Therefore, except for CPT-1A-related FAO, we analyzed that lipid synthesis or other processes might contribute to the significant improvement of QHD-L on NAFLD. Taken together, these results suggested that the regulation of hepatic FAO plays a crucial role in the alleviation of hepatic lipid accumulation in NASH. Since CPT-1A acts as a key rate-limiting enzyme in the hepatic FAO process, it may be a potential therapeutic target of QHD for reducing hepatic lipid accumulation in NASH.

Based on the above results, several pathways have been demonstrated to be associated with FAO; peroxisome proliferator-activated receptor-alpha mediates the induction of FAO in the fasted state (Bartlett and Eaton 2004; Preidis et al. 2017), AMPK relieves the allosteric inhibition of malonyl-CoA on CPT1 (Lochner et al. 2015), and STAT3 upregulates CPT-1B transcriptionally (Wang et al. 2018). Phosphorylation of JAK2/STAT3 leads to their dissociation from the receptor and the formation of active dimers, which translocate to the nucleus to regulate gene expression, thus regulating metabolism, reproduction, oxidative stress, apoptosis, and inflammation (Aaronson and Horvath 2002; Yu et al. 2014). STAT3 regulates CPT-1 expression in breast cancer stem cells by directly binding to the CPT-1 promoter, thereby reducing FAO and ATP production in breast cancer (Wang et al. 2018). In diet-induced obese mice, AG490, an inhibitor of JAK2, inhibited liver inflammation and tumour growth via STAT3 (Park et al. 2010). In HFD-fed mice, the blocking of STAT3 signalling reduced obesity and insulin resistance, further demonstrating that STAT3 is a molecular link in obesity (Priceman et al. 2013). Therefore, we evaluated the phosphorylation levels of JAK2/STAT3 in the livers of mice. Our data showed that these were upregulated after the HFHC diet, but significantly downregulated after QHD treatment. These results suggest that the effect of QHD on FAO may be related to the upregulation of CPT-1A expression via inhibition of the JAK2/STAT3 signalling pathway.

In the present study, we demonstrated that QHD regulated the lipid accumulation by increasing FAO mediated by the JAK2/STAT3/CPT-1A signalling pathway *in vivo.* However, there are some limitations in this study. Herbs and natural active ingredients in the diet, such as genistein (Xin et al. 2019) and EGCG (Chen et al. 2018), are effective in treating NAFLD (Liu et al. 2017). As studied previously, the main active components of QHD were described as following: geniposide, genipin, chlorogenic acid, resveratrol, polydatin and kaempferol. Genipin effectively regulated miR-142a-5p/SREBP-1c axis to antagonized HFD-induced hepatic lipid accumulation in mice (Zhong et al. 2018). Meanwhile, kaempferol has been reported to upregulate the expression and total activity of hepatic CPT1A (Wang et al. 2012). Active ingredients in QHD, such as geniposide and chlorogenic acid combination, have been reported to improve NAFLD by inhibiting hepatic stearoyl-CoA desaturase 1 (Chen et al. 2021). Although some active ingredients in QHD against NASH, such as geniposide and chlorogenic acid, have been reported (Chen et al. 2021), the specific active ingredients regulating JAK2/STAT3/CPT-1A-related FAO need to be further explored. The underlying mechanism needs to be further investigated *in vitro*.

## Conclusions

We found that in the NASH mice induced by HFHC diet, QHD reduced hepatic lipid accumulation by increasing FAO, and this effect might be related to the JAK2/STAT3/CPT-1A signalling pathway. This study not only facilitates the development and utilization of Chinese herbal medicines, but also, it serves as a scientific basis for the use of QHD in the treatment of NASH.

## Author contributions

FQ designed this study. SQM and WX performed the experiments. FQ, SQM, XX, WX, and AZM analyzed the data. SQM wrote the manuscript. FQ and HYY revised the manuscript. All authors critically participated in the discussion and commented on the manuscript. SQM and WX contributed equally to this work.

## Geolocation information

528 Zhangheng Road, Pudong New Area, Shanghai, China.

## Data Availability

The data used to support the findings of this study are available from the corresponding author upon request.
